# Cryopreserved leukopaks maintain cell viability and functionality: a solution for cell therapy logistics

**DOI:** 10.1186/2051-1426-3-S2-P383

**Published:** 2015-11-04

**Authors:** George Eastwood, Wenshi Wang, Tia Hexom, Laura-Marie Nucho

**Affiliations:** 1 HemaCare BioResearch Products & Services, Van Nuys, CA, USA

## Background

Human cells are critical raw materials for research and manufacturing of cell therapy products. However, accessibility to freshly procured cells can be limited, creating a crucial need for a suitable alternative to fresh cells that are viable and functional, especially when transporting materials globally.

## Methods

At HemaCare, we have investigated the viability and functionality of lymphocytes, both fresh and cryopreserved, from leukopaks (leukapheresis collections) procured within our FDA registered cGMP donor collection facility. Fresh leukopaks (LP) from healthy donors were evaluated for cell viability via flow cytometry over the course of seven days.

## Results

Studies found that viability of LPs in autologous plasma at room temperature were greater than 80% up to 144 hours post collection. However, the cell counts decreased steadily over time, leading us to look at how cryopreservation might help to ameliorate these drawbacks in typical LP transport. The viability of T cells, monocytes, B cells, and natural killer (NK) cells from LP were evaluated from the same donor pre- and post-cryopreservation. Preliminary data for whole LPs shows that post-cryopreserved viability averages 97.5% (+/- 1.2 SEM). The distribution of the CD3+, CD4+, and CD8+ populations were 43.4%, 28.7%, and 12.2%, respectively, within the total LP. Distribution of B-cells, NK cells, and monocytes shows 8.38%, 12.5%, and 20.4%, respectively. T cell functionality data was also obtained, as this cell type is sensitive to the cryopreservation process. Results of CFSE-labeled T cells functional assays show multiple divisions over 5 days, and high expression of Ki67 and CD25 after 5 day monocyte derived DC stimulation.

## Conclusions

These preliminary results suggest that cryopreserved LPs can serve as an acceptable alternative to fresh LPs. Thus, cryopreserved LPs are a valuable and significant option for emerging autologous and allogeneic cell therapies which require apheresis shipments from collection centers to cell therapy processing facilities around the world.

